# Primary Ewing sarcoma of the kidney mimicking hydatid cyst: A case report with literature review

**DOI:** 10.1016/j.radcr.2025.10.001

**Published:** 2025-11-01

**Authors:** Rebaz M. Ali, Shakhawan Hama Amin Said, Shano M. Ali, Mzhda Sahib Jaafar, Rawa Bapir, Rawa M. Ali, Hadeel Adnan Yasseen, Rezheen J. Rashid, Hiwa O. Abdullah, Sasan M. Ahmed, Fahmi H. Kakamad

**Affiliations:** a Hiwa Cancer Hospital, Sulaymaniyah Directorate of Health, Sulaymaniyah, Iraq; b Scientific Affairs Department, Smart Health Tower, Sulaymaniyah, Iraq; c College of Medicine, University of Sulaimani, Sulaymaniyah, Iraq; d Department of Urology, Sulaymaniyah Surgical Teaching Hospital, Sulaymaniyah, Iraq; e Hospital for Treatment of Victims of Chemical Weapons, Halabja, Iraq; f Department of Radiology, Hiwa Cancer Hospital, Sulaymaniyah, Iraq; g Kscien Organization for Scientific Research, Sulaymaniyah, Iraq

**Keywords:** Ewing sarcoma, Small round blue cell tumor, Primitive neuroectodermal tumor, Renal carcinoma, Nephrectomy, Chemotherapy

## Abstract

Primary renal Ewing sarcoma (EWS) is a rare and aggressive disease with a poor prognosis. Due to its nonspecific presentation and radiological findings, it can be misdiagnosed. This study reports a case of primary renal EWS mimicking a hydatid cyst. The case (31-year-old female) presented with right loin pain for a one-month duration. Abdominal magnetic resonance imaging demonstrated a well-defined, thick-walled cystic lesion occupying the upper and middle poles of the kidney, compressing the adjacent calyces and renal vessels, and showing thin peripheral septa. The features were suggestive of a hydatid cyst. The latex agglutination test for hydatid disease was negative. An excisional biopsy followed by a partial nephrectomy was performed, and histopathology confirmed the cyst as primary renal EWS. Extraosseous cases are rare, accounting for approximately 6% of all EWS cases. Primary renal EWS comprises only 1% of renal tumors, with fewer than 150 cases reported. Early detection and management of renal EWS may be challenging due to the nonspecificity of imaging features, as the disease can mimic other differential diagnoses, including hydatid cysts.

## Background

Ewing sarcoma (EWS), also known as peripheral neuroectodermal tumor, is a malignant bone tumor that mainly affects children and young adults, peaking in the second decade of life. Extraosseous cases are rare, accounting for approximately 6% of the cases. The most common sites include the head and neck regions, trunk, extremities, and retroperitoneum [1,2]. Primary renal EWS is a sporadic and aggressive condition that comprises only 1% of renal tumors [3,4]. Due to its aggressive nature, this tumor highly metastasizes, commonly to the lungs, followed by the regional lymph nodes and the liver [3]. The disease has a poor prognosis, typically with survival of less than one year. This is due to factors including delayed diagnosis, large tumor size, aggressive behavior, and high likelihood of metastasis [4,5]. Due to the nonspecific clinical presentation and radiological imaging findings of the tumor, it can be misdiagnosed provisionally [6,7]. This study aims to report a case of primary renal EWS in an adult female. The report has been written based on the CaReL guidelines [8].

## Case presentation

### Patient information

A 31-year-old female presented with right loin pain for one month without any other symptoms. Her past medical and surgical histories were negative.

### Clinical findings

Apart from right loin tenderness, no other significant abnormality was noticed during the physical examination.

### Diagnostic assessment

**Fig. 1:**
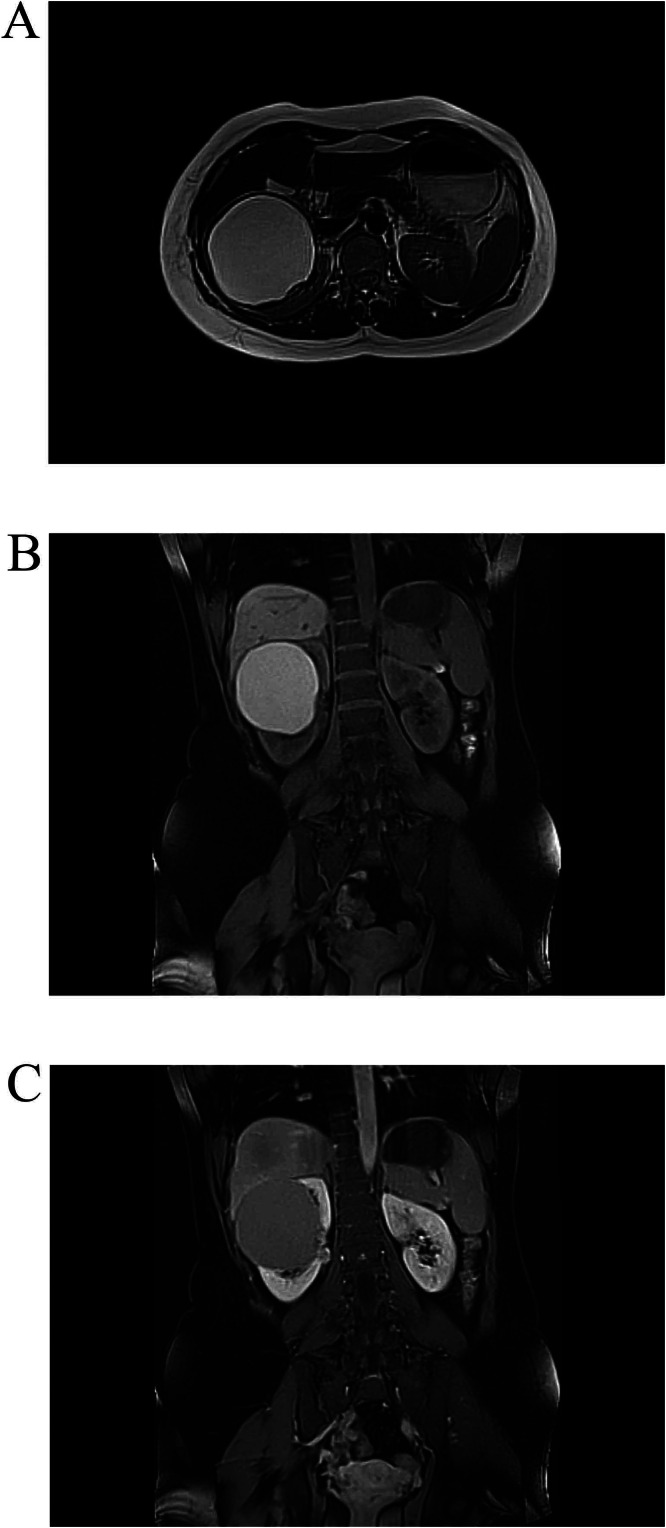
The right renal cystic lesion appeared hyperintense on axial T1WI. A) hyperintense signal on axial T2 WI. B) No signal loss on precontrast coronal fat suppression T1WI, and no restriction on DWI (not shown). C) No obvious enhancement in postcontrast coronal fat suppression T1WI.

Blood investigations, including complete blood count and liver and renal function tests, were normal. An abdominal and pelvic CT scan showed a large (9×9×9 cm), thick-walled, cystic lesion occupying most of the anterior aspect of the upper pole and midportion of the right kidney. It exerted pressure on the pelvicalyceal system and renal vein, resulting in the formation of renal collaterals. The cyst showed internal thin, enhancing septation with no mural nodules or calcification (image not available). Abdominal magnetic resonance imaging (MRI) revealed a well-defined, thick-walled cystic lesion in the upper and middle poles of the kidney (9.5×9 cm), compressing the adjacent calyces and renal vessels, and exhibiting thin peripheral septa with hyperintense signals on T1 and T2 images. The cyst showed no signs of restricted diffusion, enhancing septa, or solid components (Fig. 1). The features were suggestive of a hydatid cyst. However, the cyst lacked daughter cysts, floating membranes, or peripheral calcification of the cyst wall. The latex agglutination test for hydatid disease was negative.

### Therapeutic interventions

**Fig. 2:**
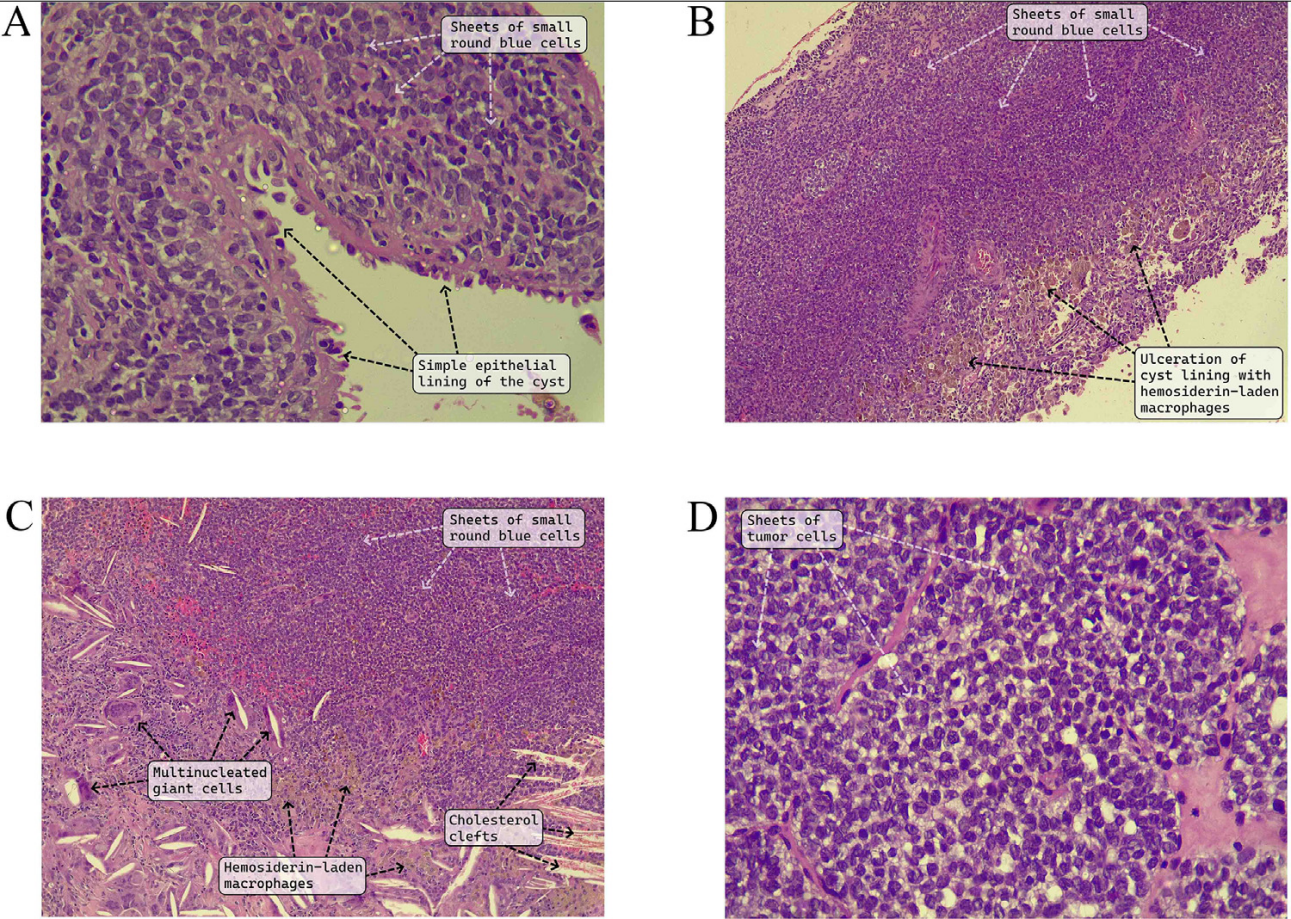
A) The renal cyst has a simple epithelial lining of cuboidal and low columnar cells with underlying solid nodules composed of sheets of small, round, blue cells. B) The cyst lining shows extensive ulceration with replacement by sheets of hemosiderin-laden macrophages. C) There are cholesterol clefts engulfed by multinucleated giant cells. D) The tumor cells are small to medium-sized and have scant, lightly eosinophilic to clear cytoplasm with round to oval, hyperchromatic nuclei that have irregular nuclear outlines and occasional grooves. (Hematoxylin and eosin [A-D]; original magnification x 400 [A and D], x 100 [B and C]).

An excisional biopsy followed by a partial nephrectomy without lymph node dissection was performed. Histopathologic examination showed a cyst lined by a simple cuboidal epithelium with bland, round nuclei. The wall of the cyst contained sheets of monomorphic, small cells with indistinct cytoplasmic membranes, scant clear or lightly eosinophilic cytoplasm, and round nuclei with stippled chromatin, nuclear membrane irregularity, and occasional grooves. Mitotic activity was low (4 to 5/ per 10 high-power fields), and there was no coagulative tumor necrosis. The cyst lining was ulcerated in areas with sheets of hemosiderin-laden macrophages and cholesterol clefts engulfed by multinucleated giant cells. The resection margin was free from tumor (Fig. 2). Immunohistochemistry (IHC) showed positivity of the tumor cells for CD99 (cytoplasmic staining of moderate intensity in >50% of the tumor cells) and FLI-1 (nuclear staining of moderate intensity in >50% of the tumor cells). AE1/AE3 highlighted the epithelial lining of the cyst, but was negative in the tumor cells. INI-1 was retained in the tumor cells, and WT1, desmin, myogenin, BCOR, TLE-1, CD45CLA, and synaptophysin were all negative (not shown). The combined histologic and immunohistochemical findings were consistent with a primary EWS of the kidney. The patient received 12 cycles of adjuvant chemotherapy (vincristine, actinomycin D, cyclophosphamide/ ifosfamide, and etoposide {VAC/IE}) every 21 days; each cycle was received over five days.

### Follow-up and outcome

**Fig. 3:**
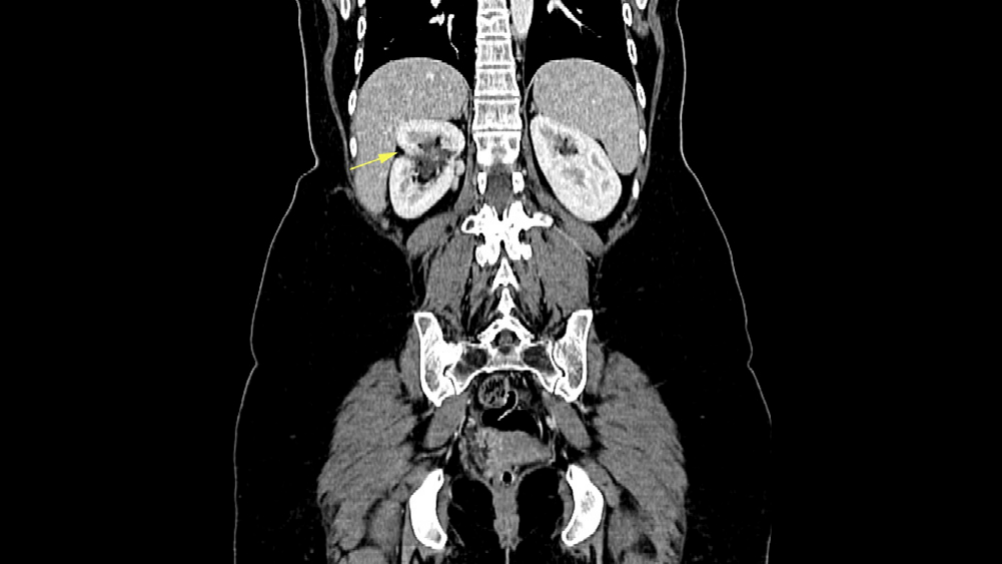
Coronal reformatted contrast-enhanced abdominal CT shows signs of operation at the mid portion in the form of renal cortical discontinuity (arrow).

Follow-up included abdominal and pelvic CT scans (Fig. 3) every three months and two positron emission tomography (PET) scans, all of which were normal. However, the third PET scan, taken approximately one year postoperation, revealed an FDG-avid right paracolic peritoneal nodule (1.3×0.9 cm, SUV max 7.1), suspicious for metastasis (not shown). Excision of the peritoneal surface nodule in the paracolic gutter was performed along with an omental biopsy and a peritoneal wash aspirate of the right paracolic gutter. The peritoneal surface nodule exhibited chronic inflammation with foreign body-type granulomas, indicating no malignancy. The omental biopsy and peritoneal wash aspirate also revealed no significant pathology and were negative for malignancy. After one year of follow-up, the patient remains disease-free.

## Discussion

EWS was first identified in 1918 and is classified within the family of small round-cell tumors [2]. However, the first case of renal EWS was reported in 1975, and since then, fewer than 150 cases have been reported in the literature [3,7]. The tumor arises from neural crest cells or mesenchymal stem cells. It is characterized by the t(11;22)(q24;q12) translocation, present in 85% to 95% of the cases, which translocates the EWSR1 gene on chromosome 22 to the FLI-1 gene on chromosome 11 [3,9].

The mean age at diagnosis has been reported to be 24.9 years, with a male predominance. Patients commonly present with flank pain, hematuria, and symptoms related to urinary tract infections. Hematuria may result from tumor infiltration into the renal collecting system [2,3]. Risi et al. reviewed 116 cases of renal EWS and reported the most common clinical symptoms as pain (54%), hematuria (29%), and renal mass (28%) [10]. Approximately 66% of patients present with distant metastasis, with the lungs being the most common site, followed by the liver and bones. Upon reviewing 46 cases of renal EWS in this study [2,3,6,7,[11], [12], [13], [14], [15], [16], [17], [18], [19], [20], [21]] (Table 1, Table 2), the mean age was found to be 28.8 years, without a gender predilection, which is slightly different from the findings mentioned previously. In line with the Risi et al. study [10], the prevalent symptoms were flank pain (45.6%), followed by hematuria (34.8%), abdominal pain (15.2%), and a palpable mass (10.9%). In 45 cases (98%), the involvement was unilateral, while it was undetermined in the other case. Both sides were equally affected, with a mean mass size of 11 cm. In total, 30 cases (65%) had metastasis, consistent with the literature, with the lung being the most commonly involved organ (45.7%), followed by bone/bone marrow (24%) and lymph nodes (15.2%). The present case was a female (31 years old) who presented with right loin pain for a month without any other symptoms.

**Table 1:** Review of 46 cases of primary renal Ewing sarcoma.

Author/ reference	Age (year)/ Sex	MCC	Imaging findings	Tumor size (cm)	DFD	MET	Management	Follow up
Aithal et al. [2]	40/F	Hematuria	An enhancing lesion extending into the renal hilum, showing vein thrombosis	5.7	RCC	No	RNT	N/A
Khudair et al. [3]	38/F	Abdominal pain, Constipation	A large heterogeneous noncalcific exophytic mass	20	RCC	Lung, lymph nodes	VDC, MESNA, IE.	Died
El Mohtarim et al. [11]	14/F	Abdominal pain and swelling, anorexia, weight loss	A large lobulated retroperitoneal tumor	16.5	N/A	Lung, lymph nodes	VAC, IE, RNT	No recurrence after 6 months
Bray et al. [12]	31/F	Hematuria, flank pain	Heterogeneous, solid, echogenic mass with internal vascularity	12	RCC or Angiomyolipoma	No	RNT, VDC, IE	No recurrence after 12 months
Ilhan et al. [13]	54/M	Hematuria, flank pain	Heterogeneously enhanced hyperdense cystic mass lesion	7	RCC	No	RNT, VAC, IE	No recurrence
Patra et al. [14]	33/F	Abdominal pain	Heterogenous mass with hemorrhage and necrosis	7.4	N/A	Lung, bowel serosa	RNT, CT	Died
	35/M	Palpable lump		4.5	N/A	No	RNT, CT	No recurrence
	19/M	Palpable lump, hematuria		19	N/A	Liver	VDC, etoposide, RNT	LWD
	28/M	Abdominal pain		6.4	N/A	Bone	VDC, etoposide, RNT	LWD
Sardana et al. [7]	49/M	Hematuria, flank pain	A lobulated mass	6.3	RCC	No	RNT, CT	No recurrence
Alahmadi et al. [6]	16/M	Hematuria, flank pain	A large heterogeneous mass	15	RCC/ Wilms tumor/sarcoma	No	RNT, etoposide, MESNA, VDC	No recurrence
Bradford et al. [15]	16/M	Abdominal pain	N/A	N/A	Wilms tumor	Lung, bone	PNT, vincristine, actinomycin, VDC, IE, irinotecan, temozolomide	Died
	11/M	Flank and testicle pain	A large mass	N/A	N/A	N/A	RNT, VDC,IE,busulfan,melphalan,thiotepa, Everolimus, VIT, VTC	Recurred after 60 months
	18/F	Hematuria, flank pain	N/A	7.5	N/A	Lung	RNT, VDC, IE	No recurrence after 56 months
	17/F	Kidney disease	N/A	N/A	N/A	No	VDC, IE	No recurrence after 70 months
	16/F	Renal mass	N/A	N/A	N/A	Lung	VDC, IE, RNT	No recurrence after 22 months
	13/F	Abdominal and back pain, lower extremity neuralgia, neurogenic bladder, ataxia.	A large heterogeneous mass	N/A	N/A	Bone, bone marrow, lungs, liver	VDC, IE, RNT, ureterectomy	Died
	15/F	Abdominal and back pain, ataxia	A large renal mass, complete occlusion of the inferior vena cava, bilateral pulmonary metastasis	N/A	N/A	Lung, bone marrow	RNT, partial ureterecomy,VDC, IE, cyclophosphamide	Recurred after 6 months, and the case died after 30 months from presentation
Cheng et al. [16]	31/F	Flank pain, abdominal mass	A hypoechoic mass with central necrosis	15.4	N/A	Lymph node, neck, adrenal gland, psoas major, diaphragm, lung	RNT, VAC,IE, carboplatin, antiangiogenic drug, apatinib	No recurrence after 18 months
Doroudinia et al. [17]	27/F	Hematuria, flank pain	N/A	7.3	Sarcomatous tumor	No	RNT, VDC, IE	No recurrence
Suzuki et al. [18]	45/F	Abdominal pain	A renal mass with contrast enhancement	9.8	RCC	No	PNT	No recurrence after 12 months
Murugan et al. [19]^a^	41/M	Flank pain	N/A	12	N/A	Meninges, bone	CT	Died
	35/M	Flank pain	N/A	17	N/A	Lung, lymph nodes	RNT, CT	Died
	43/M	Flank pain	N/A	20	N/A	Bone, lung	CT	Died
	33/M	Flank pain	N/A	N/A	N/A	Lung, brain, liver, skin	RNT, CT	Died
	70/F	Flank pain	N/A	N/A	N/A	Lung	RNT, CT, INF	Died
	21/M	Flank pain	N/A	7.2	N/A	Peritoneum	RNT, CT	Died
	23/M	Flank pain	N/A	9	N/A	Lung	RNT, CT	Died
	45/M	Flank pain	N/A	N/A	N/A	Adrenal gland, lymph nodes	RNT, CT	Died
	31/F	Flank pain	N/A	12.5	N/A	No	RNT, CT	No recurrence after 108 months
	32/F	Flank pain	N/A	12	N/A	Lung, lymph nodes	RNT, CT	Died
	50/M	Flank pain	N/A	N/A	N/A	N/A	N/A	N/A
	52/F	Flank pain	N/A	N/A	N/A	Lung, liver	RNT, CT, INF	Died
	25/M	Flank pain	N/A	11.4	N/A	Lung	RNT, CT	Died
	26/F	Hematuria	N/A	11	N/A	No	RNT, CT	No recurrence after 48 months
	29/M	Hematuria	N/A	9.2	N/A	Lung	RNT, CT	N/A
	8/M	Hematuria	N/A	N/A	N/A	Bone	RNT, CT	N/A
	9/F	Hematuria	N/A	19	N/A	Bone	RNT, CT, RT	N/A
	18/F	Hematuria	N/A	5	N/A	Bone, lymph node, brain	RNT, CT, RT	LWD
	33/F	Hematuria	N/A	8.5	N/A	Lung	RNT, CT	N/A
	32/M	Hematuria	N/A	15	N/A	No	RNT, CT	No recurrence after 14 months
	19/M	Hematuria	N/A	6	N/A	Lung, bone	RNT, CT, RT	Died
	33/F	Palpable mass	N/A	N/A	N/A	Lung	RNT, CT	Died
	24/F	Palpable mass	N/A	14	N/A	No	RNT, CT	No recurrence after 156 months
Sadiq et al. [20]	14/F	Flank pain	A large heterogeneous mass with a large necrotic component	8	N/A	Abdominal wall	Nonradical nephrectomy, VDC, IE	N/A
Yoshihara et al. [21]	14/F	Abdominal pain	A mass with contiguous extension through the renal vein into the inferior vena cava	8.9	Wilms’ tumor, clear cell sarcoma, RCC	No	RNT, VDC, IE	No recurrence after 70 months

MCC, main chief complaint; N/A, nonavailable; DFD, differential diagnosis; RCC, renal cell carcinoma; RNT, radical nephrectomy; VAC, (vincristine, actinomycin D, cyclophosphamide); VDC, (vincristine, doxorubicin, cyclophosphamide); IE, (ifosfamide, etoposide); MESNA, Sodium 2-mercaptoethane sulfonate; MET, metastasis; LWD; live with disease; NACT, neoadjuvant chemotherapy; CT, chemotherapy (unknown type); PNT, partial nephrectomy; VIT, (vincristine, irinotecan, and temozolomide); VTC, (vincristine, topotecan, and cyclophosphamide); INF, interferon therapy; RT, radiotherapy.

a The symptoms for that study were randomly distributed among the cases as they were not specified for each case in the original study.

**Table 2:** Summary of the reviewed cases.

Variables	Frequency / Percentage
Patient demographics	
Age (mean ± SD)	28.8 ± 13.5
Sex	
Male	21 (46.0%)
Female	25 (54.0%)
Main chief compliant/ symptoms^a^	
Flank pain	21 (45.6%)
Hematuria	16 (34.8%)
Abdominal pain	7 (15.2%)
Palpable mass	5 (10.9%)
Others	12 (26%)
Laterality	
Right	23 (50.0%)
Left	22 (48.0%)
N/A	1 (2.0%)
Tumor size, range (mean ± SD) cm	4.5 – 20 (11 ± 4.6)
Differential diagnosis^a^	
Renal cell carcinoma	8 (17.4%)
Wilms tumor	3 (6.5%)
Sarcoma	3 (6.5%)
Angiomyolipoma	1 (2.0%)
Metastatic cases^a^	
Lung	21 (45.7%)
Bone/bone marrow	11 (24%)
Lymph nodes	7 (15.2%)
Liver	3 (6.5%)
Brain	2 (4.3%)
Adrenal gland	2 (4.3%)
Others	8 (17.4%)
Management	
Nephrectomy alone	2 (4.3%)
Nephrectomy with chemotherapy	39 (85.0%)
Chemotherapy alone	4 (8.7%)
N/A	1 (2.0%)
Positive immunohistochemistry^a^	
CD99	35 (76.0%)
FLI-1	10 (21.7%)
NKX 2-2	7 (15.2%)
Synaptophysin	6 (13.0%)
Vimentin	4 (8.7%)
Others	8 (17.4%)
Follow up	
Alive	21 (45.7%)
Died	18 (39.1%)
N/A	7 (15.2%)
Recurrence	2 (4.3%)

SD, standard deviation; N/A, nonavailable.

a In each case, multiple options for each marker may be present.

Imaging should not be the primary diagnostic tool for renal EWS due to the lack of specific imaging features, which can result in misdiagnosis. Instead, greater emphasis should be placed on histopathology, IHC, and cytogenetic studies to confirm the diagnosis. Hence, most cases are diagnosed postoperatively [3]. Microscopically, most cases are characterized by uniform small round cells with round nuclei, finely stippled chromatin, inconspicuous nucleoli, minimal clear or eosinophilic cytoplasm, and indistinct cytoplasmic membranes [11]. The common differential diagnoses for renal EWS include Wilms tumor, neuroblastoma, clear cell sarcoma, lymphoma, rhabdomyosarcoma, the small cell variant of osteosarcoma, desmoplastic small round cell tumor, small cell neuroendocrine carcinoma, and nephroblastoma. The tumor cells may be arranged in Homer-Wright rosettes, and IHC often shows strong positivity for CD99 and FLI-1. New markers, such as NKX2.2, have further improved diagnostic accuracy [2,11]. NKX2.2 is a protein involved in regulating gene expression within the neuroendocrine and glial differentiation pathway. NKX2.2 is a specific marker for targeting the fusion protein EWS-FLI-1, demonstrating a high sensitivity of 93% and specificity of 89%. However, fluorescent in situ hybridization (FISH) represents the gold standard method, exhibiting high sensitivity (92.3%) and specificity (100%) [11]. Among the reviewed cases, the tumors in 10 cases were preoperatively suspected to be either renal cell carcinoma (17.4%), Wilms tumor (6.5%), sarcoma (6.5%), or angiomyolipoma (2%). In the present case, the MRI features of the cyst (9 cm) were suggestive of a hydatid cyst rather than a complicated cyst. It was a thick-walled cystic lesion with thin peripheral septa; however, the cyst lacked daughter cysts, floating membranes, or peripheral calcification of the cyst wall. The echinococcal serological test was negative.

There is no consensus on the optimal treatment for renal EWS. However, the standard approach involves a multimodal strategy comprising surgical resection, nephrectomy, chemotherapy, and radiotherapy. Chemotherapy typically alternates between VDC (vincristine, doxorubicin, cyclophosphamide) and IE (ifosfamide, etoposide) regimens. This alternating regimen has been shown to enhance outcomes in nonmetastatic EWS [3]. Nevertheless, this regimen is linked to several hematological and nonhematological toxicities. Grade 3 and 4 hematological toxicities, including anemia, thrombocytopenia, and neutropenia, are frequent. Nonhematological toxicities include bacteremia, urinary tract infections, mucositis, and fever [22]. Furthermore, if venous thrombosis is present alongside pulmonary metastasis, cavotomy may be incorporated into the surgical procedure. When surgical margins are positive or there is localized lymph node involvement, radiotherapy should be administered as salvage therapy. However, opinions differ regarding radiotherapy as the first-line treatment option [13]. The prognosis of EWS remains poor, especially if metastasis is present, with a cure rate of only 20% and an overall 5-year survival [2,23]. In our review, 85% of the cases were managed with a combination of nephrectomy and chemotherapy. Only six cases (13%) underwent a single modality of therapy (8.7% chemotherapy alone and 4.3% nephrectomy alone). The IHC staining was positive for CD99 in 76% of the cases, FLI-1 in 21.7%, NKX2-2 in 15.2%, synaptophysin in 13%, and vimentin in 8.7%. Among the cases with a known prognosis (84.8%), nearly half of the cases (39.1%) died due to the disease. Recurrence was found in two cases: one died, and the other’s destination was unknown. As there were no signs of metastasis on the imaging findings of the present study, the patient underwent an excisional biopsy followed by a partial nephrectomy without a lymph node dissection. A diagnosis of primary renal EWS was made on the excisional specimen. The IHC staining was positive for CD99 and FLI-1 (in >50% of tumor cells) and negative for AE1/AE3, INI-1 loss, WT1, desmin, myogenin, BCOR, TLE-1, CD45CLA, and synaptophysin. The patient received 21 cycles of VAC/IE without side effects. The case remains disease-free with no signs of recurrence or metastasis after one year of follow-up. This report's limitation is the absence of a FISH test due to its high cost to the patient.

## Conclusion

Primary renal EWS is a rare disease with a poor prognosis. Early detection and management may increase the chances of preventing metastasis and enhancing overall survival. However, imaging findings may not be reliable for an accurate diagnosis, as the disease can mimic a hydatid cyst, among other common differential diagnoses. For radiologists, any large, atypical cystic renal lesion in a young patient that does not entirely fit benign or infectious patterns, should raise suspicion for rare primary renal tumors, including sarcomas, and necessitate multidisciplinary evaluation.

## Patient consent

Informed consent was taken from the patient to publish any accompanying data and images.
